# Insights into the Functional Role of ADTRP (Androgen-Dependent TFPI-Regulating Protein) in Health and Disease

**DOI:** 10.3390/ijms22094451

**Published:** 2021-04-24

**Authors:** Cristina Lupu, Maulin M. Patel, Florea Lupu

**Affiliations:** 1 Cardiovascular Biology Research Program, Oklahoma Medical Research Foundation, Oklahoma City, OK 73104, USA; Florea-Lupu@omrf.org; 2 Department of Cell Biology, University of Oklahoma Health Sciences Center, Oklahoma City, OK 73104, USA; maulin-patel@ouhsc.edu

**Keywords:** ADTRP, TFPI, cardiovascular disease, coagulation, FAHFA

## Abstract

The novel protein ADTRP, identified and described by us in 2011, is androgen-inducible and regulates the expression and activity of Tissue Factor Pathway Inhibitor, the major inhibitor of the Tissue Factor-dependent pathway of coagulation on endothelial cells. Single-nucleotide polymorphisms in ADTRP associate with coronary artery disease and myocardial infarction, and deep vein thrombosis/venous thromboembolism. Some athero-protective effects of androgen could exert through up-regulation of *ADTRP* expression. We discovered a critical role of ADTRP in vascular development and vessel integrity and function, manifested through Wnt signaling-dependent regulation of matrix metalloproteinase-9. ADTRP also hydrolyses fatty acid esters of hydroxy-fatty acids, which have anti-diabetic and anti-inflammatory effects and can control metabolic disorders. Here we summarize and analyze the knowledge on ADTRP and try to decipher its functions in health and disease.

## 1. General Introduction

The novel protein ADTRP (androgen-dependent TFPI-regulating protein), identified and described by Lupu and colleagues in 2011, is androgen-inducible and up-regulates the expression and activity of tissue factor pathway inhibitor (TFPI), the major inhibitor of the tissue factor (TF)-dependent pathway of coagulation on endothelial cells (EC). Endothelial dysfunction, including dysregulated production and activity of the enzymes and/or inhibitors that control coagulation and fibrinolysis, impact vascular homeostasis. This favors the development of pathologies involving disruption of the vascular wall, such as myocardial infarction (MI), deep vein thrombosis (DVT)/venous thromboembolism (VTE), sepsis, and cancer.

Along the ten years from its discovery, a few labs tried to address the physio-pathologic role of ADTRP and a variety of potential functions emerged for ADTRP distinct from TFPI regulation. Many pieces of evidence suggest that ADTRP is potentially beneficial in preventing cardiovascular disease (CVD). Nevertheless, the heterogeneity of roles attributed to ADTRP and the lack of mechanistic insights make it hard to connect them all into a unitary functional model at this moment. This review is aimed to bring together all the present knowledge about ADTRP in an attempt to open the field for the development of mechanistic studies designed to decipher the main function(s) of ADTRP in health and disease.

## 2. Discovery of ADTRP

TFPI is a key natural inhibitor of coagulation: it neutralizes factor (F) Xa and inhibits TF-FVIIa in the presence of FXa, thus precluding thrombin generation. Thrombin performs a number of procoagulant functions, including the activation of platelets, FV, FVIII and FXI, and cleavage of fibrinogen to fibrin.

Low plasma levels of TFPIα (full-length TFPI) correlate with prothrombotic diseases, including DVT/VTE [1], coronary artery disease (CAD), ischemic stroke, peripheral artery occlusive disease (reviewed in [2]), and, lately, COVID-19 [3]. Excessive and/or ectopic TF-driven coagulation not adequately balanced by TFPI [4,5,6] could lead to thrombotic complications in sepsis [7], atherosclerosis [4,8], lupus [9] and cancer [10].

Discovery of the role of TFPI in bleeding disorders [11,12] has helped us to understand the interdependent relationship between the procoagulant and anticoagulant mechanisms of hemostasis.

On EC, TFPIβ is glycosyl phosphatidylinositol (GPI)-anchored to the plasma membrane in caveolae/lipid rafts [13,14,15,16]. TFPI is expressed primarily in EC and megakaryocytes [14,17,18], but also in monocytes and smooth muscle cells (SMC) [4,6]. The GPI-anchored TFPIβ represents the most physiologically significant inhibitor of TF-FVIIa and FXa [14,15,19,20,21]. Platelets contain TFPIα, which is essential for the inhibition of early forms of prothrombinase, which precludes excessive thrombin generation in the initial stages of clot formation [22].

Despite TFPI being a major endogenous inhibitor of coagulation, very few mechanisms/factors that regulate the natural expression of TFPI have been identified so far [23,24,25]. Data concerning the effects of androgens on TFPI in vitro [26,27,28,29] and in patients [30,31] suggest a beneficial role of androgens in cardiovascular function, but the causality of this association has not been proven. We considered it important to discover new ways to regulate the expression and function of cell-associated TFPI to prevent unwanted clotting and inhibit TF-dependent pathologies. For this purpose, we applied the method developed by Wren [32], namely the global meta-analysis (GAMMA) of over 3500 NCBI’s Gene Expression Omnibus (GEO) 2-channel human microarray datasets and we identified C6ORF105 as having a very high score of co-expression with TFPI [28].

Experimentally, we verified that the uncharacterized protein encoded by *C6ORF105* regulates TFPI mRNA expression, cellular distribution and EC-associated anticoagulant activity of the inhibitor, both in native conditions and in response to androgen, thus also acting as a novel androgen-controlled membrane protein. We named this protein ADTRP: androgen-dependent TFPI-regulating protein.

## 3. Structure, Expression, and Tissue Localization of ADTRP

Based on sequence homology, ADTRP belongs to the androgen-inducible gene family, of which androgen-inducible gene 1 (AIG1) was cloned from human dermal papilla [33] and is homologous to the hamster FAR-17a [34].

**Figure 1:**
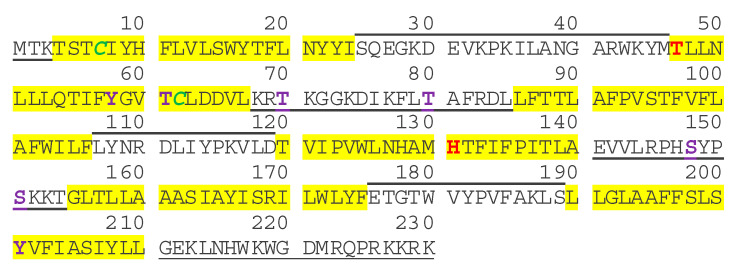
ADTRP isoform 1 (canonical) sequence and domains. According to UniProtKB/SwissProt, the protein Q96IZ2-1 has six predicted transmembrane domains (yellow highlight). Cytoplasmic domains are underlined; extracellular domains are over-lined. Threonine (**T_47_**) and Histidine (**H_131_**) represent sites for FAHFA (fatty acid esters of hydroxy fatty acids) hydrolase activity. Predicted palmitoylation: **C_7_** and **C_62_**; potential phosphorylation sites: **S_148_**, **S_151_**, **T_61_**, **T_70_**, **T_80_**, **Y_58_**, and **Y_201_**.

According to UniProtKB/SwissProt (https://www.uniprot.org, accessed on 20 February 2021) *C6ORF105* encodes Protein Q96IZ2. UniProtKB lists 3 described isoforms and 4 potential isoforms that are computationally mapped. Isoform 1 (Q96IZ2-1) has been chosen as the canonical sequence with 230 aminoacids and a molecular weight of 27 kDa. The protein has six predicted transmembrane domains marked as yellow highlight on the sequence (Figure 1). Cytoplasmic domains are under-lined and extracellular domains are over-lined. Threonine (T_47_) and Histidine (H_131_) are FAHFA (fatty acid esters of hydroxy fatty acids) hydrolase activity sites [35]. Post-translational modifications predictions include two palmitoylation sites possible at cysteine residues C_7_ and C_62_; no N-glycosylation sites, but several O-glycosylation sites possible; and, phosphorylation possible at serine residues S_148_ and S_151_, as well as threonine residues T_61_, T_70_ and T_80_, and tyrosine residues Y_58_ and Y_201_ (Figure 1).

ADTRP mRNA and protein expression were detected in human EC in culture, human placenta and baboon lung and aorta [28], human mesenchymal stem cells (MSC) in culture [36] (and our results, see below), human monocytes and macrophages [37], and several human cell lines (HepG2, HEK293, HUVEC, EA.hy926, HeLa). According to GENEVESTIGATOR^®^ (https://genevestigator.com, accessed on 2 March 2021), the expression of ADTRP increases during embryogenesis and then constantly into adulthood, to be followed then by abrupt decrease with aging. Whether or not this decrease correlates with the decline in androgen levels during old age remains to be determined.

**Figure 2:**
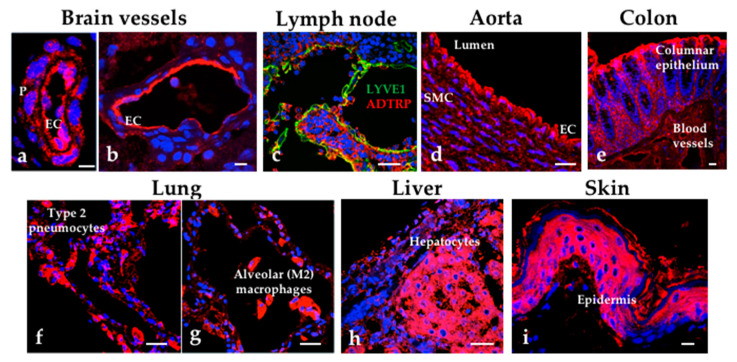
Immunofluorescence (IMF) staining for ADTRP in human tissues. IMF and confocal microscopy show the localization of ADTRP in blood vessels in the brain (**a**,**b**), lymphatic vessels (**c**), aorta (**d**), epithelium in the colon (**e**), lung (**f**,**g**) hepatocytes (**h**) and epidermis (**i**). Tissue sections (Tissue Array, Cat. No: T8234709-5, BioChain Institute Inc., Newark, CA, USA) were immunostained for ADTRP (all panels; Cy3, red) and Lyve-1 (panel c; FITC, green; marker for lymphatics) as described [28]. Blue: nuclei. EC: endothelial cells. P: pericytes. SMC: smooth muscle cells. Bars: 20 µm (**a**,**b**,**i**), 50 µm (**c**–**h**).

By immunostaining, we identified ADTRP expression in human EC in both arteries and veins, lymphatics, epithelia of various organs (lung, skin, intestine), hepatocytes, and lung alveolar macrophages (Figure 2). Wang and colleagues [38] showed that C6ORF105 was expressed in the heart, stomach, skin, and kidney.

**Figure 3:**
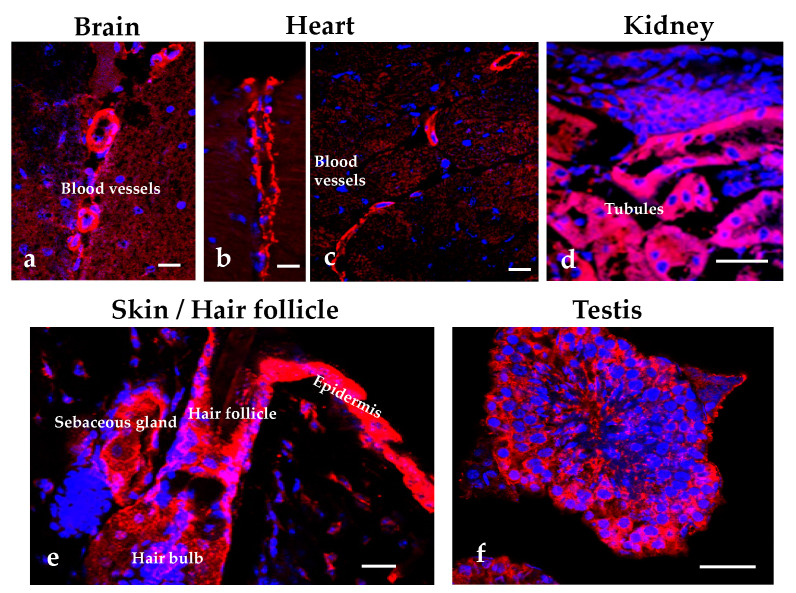
Immunofluorescence staining for ADTRP in mouse tissues. IMF and confocal microscopy illustrate the localization of ADTRP (Cy3, red) in blood vessels of the brain (**a**) and heart (**b**,**c**), kidney tubules (**d**), epidermis and hair follicle (**e**) and testis (**f**). Blue: nuclei. Bars: 50 µm.

In mouse tissues, we detected ADTRP expression by qPCR, Western blot, immunostaining and in-situ hybridization in the skin and isolated dermal fibroblasts, lung and aorta [39], as well as liver, brain, kidney, white and brown adipose tissue, intestine, and testis. Figure 3 illustrates examples of ADTRP localization in mouse tissues. The mRNA expression of Adtrp increased significantly (3–4 times) during embryogenesis [39]. Another group also reported localization of mouse ADTRP in the liver, adipose tissue, kidney, and duodenum [40]. 

In zebrafish, we showed by in-situ hybridization that *adtrp* is expressed in the lateral plate mesoderm, most likely in angioblasts, and in both the dorsal aorta and cardinal vein [39]. qPCR revealed that the mRNA expression of *adtrp* increased up to 50 times during zebrafish embryonic development and into adulthood [39].

**Figure 4:**
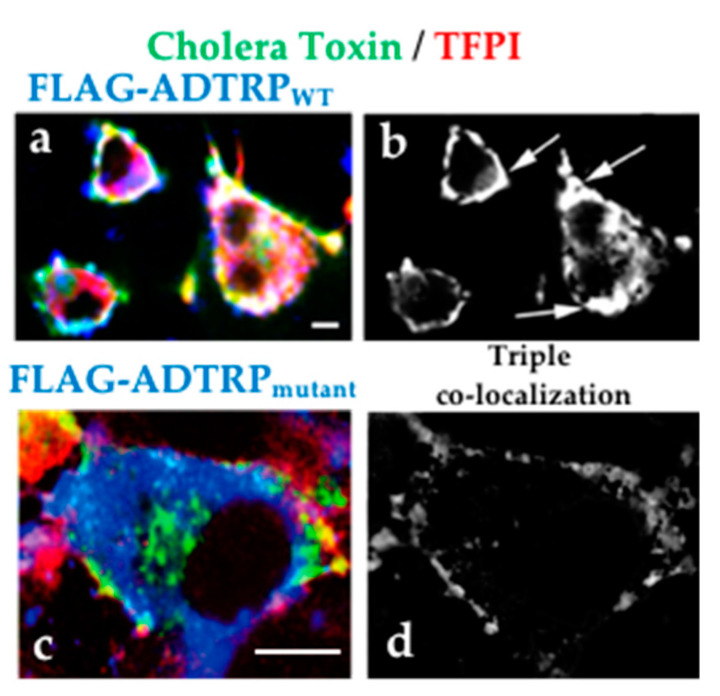
Localization of ADTRP in lipid rafts. IMF and confocal microscopy show clustering and strong overlap (**a**,**b**, triple co-localization, arrows) of cell surface TFPI (Cy3, red) and FLAG-ADTRPwt (Cy5, blue) together with Cholera Toxin-FITC (green) on EA.hy926 cells expressing FLAG-ADTRP. Panels (**c**,**d**): FLAG-ADTRPmutant has predominant cytoplasmic location and shows less overlap with TFPI and Cholera Toxin (**d**). Bars: 10 µm.

Analysis of the natural expression of ADTRP revealed that it colocalizes with both TFPI and caveolin-1 [28] in the plasma membrane of EC in culture. Similar to TFPI, ADTRP also partially resides in caveolae/lipid rafts. Both ADTRP and TFPI partition in the detergent-soluble fraction containing hydrophobic proteins after Triton X-114 extraction, and cluster together on the cell surface during live cell incubation with the lipid raft ligand Cholera Toxin [28]. The potential palmitoylation sites C_7_ and C_62_, and the trans-membrane domains could play a role in targeting ADTRP to lipid rafts/caveolae. To verify the role of palmitoylation, we mutated C_62_ residue into A and expressed the FLAG-ADTRP_mut_ construct in EC. Western blot after Triton X-114 extraction revealed decreased partition of ADTRP_mut_ in the detergent fraction. Immunofluorescence illustrates less cell surface clustering, decreased membrane co- localization of TFPI and FLAG-ADTRP_mut_ after treatment with Cholera Toxin and increased cytoplasmic localization of FLAG-ADTRP_mut_ (Figure 4). Altogether, these data suggest that palmitoylation of ADTRP supports, at least in part, its membrane localization in lipid rafts/caveolae and potential function as lipid raft domain organizer. 

Intriguingly, a very recent report detected soluble ADTRP in human plasma [41]. This is surprising considering that ADTRP has a transmembrane localization, and the authors did not address any potential mechanism for their findings.

## 4. Cross-Talk between TFPI and ADTRP

### 4.1. Regulation of ADTRP Expression by Androgen

Using prediction algorithms, we described potential half-AREs (androgen-response elements, AGAACA and TGTTCT) in the ADTRP promoter [28]. More recently, Luo and colleagues [42] confirmed experimentally that the transcriptional activation of ADTRP by androgen occurs through direct binding of the androgen receptor to the half-ARE (TGTTCT) in the ADTRP promoter/regulatory region. Using mutagenesis, they demonstrated that the half-ARE is critical for the transcriptional activation of ADTRP by testosterone.

### 4.2. Regulation of TFPI Expression by ADTRP

Luo and colleagues [43] employed luciferase reporter assays, chromatin immunoprecipitation, and electrophoretic mobility shift assay in combination with deletions of the TFPI promoter/regulatory region to identify the transcription factor POU1F1 as the key regulator of TFPI mRNA expression by ADTRP. The ADTRP-response element is localized between −806-bp and −756-bp upstream of *TFPI* transcription start site, which contains a binding site for POU1F1. Deletion of POU1F1-binding site or knockdown of *POU1F1* expression abolished ADTRP-mediated transcription of *TFPI* [43]. The precise mechanism through which ADTRP protein would gain access into the nucleus and exert transcriptional regulation of TFPI was not addressed. Nevertheless, these findings could bare potential significance in the development of strategies to reduce TFPI expression and activity and therefore bleeding in patients with hemophilia [44].

### 4.3. Functional Significance of ADTRP-TFPI Interactions

#### 4.3.1. ADTRP Regulates the Expression and Activity of Cell-Associated TFPI

Using post-transcriptional silencing and over-expression experiments in EC in vitro, we unveiled the function of novel at the time protein ADTRP in regulating the TFPI-dependent anticoagulant properties of the endothelium in both normal conditions and in response to androgen [28]. We found that ADTRP could regulate TFPI mRNA expression, cell membrane distribution, and cell-associated activity of TFPI against TF-FVIIa-dependent FX activation.

**Figure 5:**
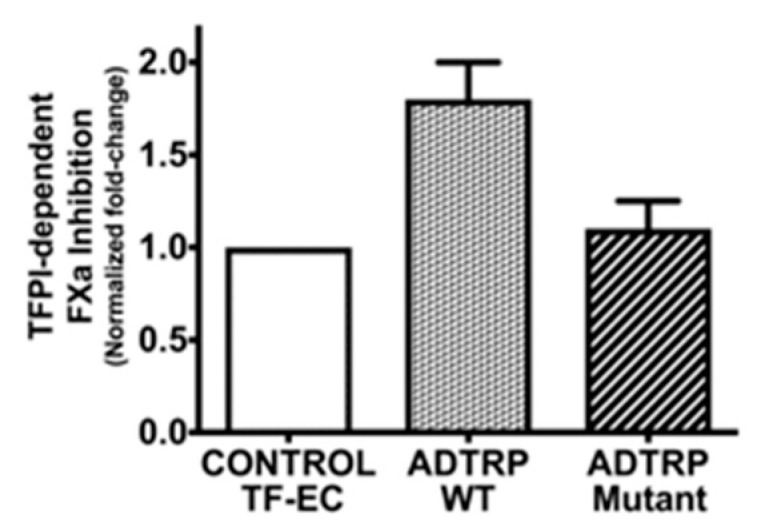
Effect of membrane-bound ADTRP on TFPI anticoagulant activity. The inhibitory function of cell surface TFPI against FX activation by TF-FVIIa was measured in the EC line EA.hy926 stable expressing low levels of TF-YFP [45] (TF-EC) as well as FLAG-ADTRP, as described [28]. The palmitoylation deficient ADTRPmutant failed to increase TFPI anticoagulant activity as compared to ADTRPwt.

Confirming the importance of ADTRP localization in lipid rafts, the palmitoylation- deficient ADTRP_mut_ failed to increase TFPI anticoagulant activity (Figure 5). 

The response to androgen required both ADTRP and caveolin-1, with androgen- mediated increase of ADTRP association with lipid rafts/caveolae being a potential key regulator of the distribution and activity of TFPI [28]. We unveiled novel mechanisms of preserving vascular homeostasis and potentially providing anticoagulant protection in pathologies like CAD, DVT/VTE, sepsis, or cancer, which may associate with pro-thrombotic states. 

#### 4.3.2. ADTRP-TFPI Axis Regulates Myelopoiesis and Hematopoiesis 

Wang and colleagues [46] demonstrated that *adtrp1*, one of the two paralogues of zebrafish *adtrp*, could regulate the specification of hemangioblasts, primitive myelopoiesis, and definitive hematopoiesis. According to the NCBI-2013 database, *adtrp1* (*zgc112175*) is the only gene homologous to human ADTRP [39]. Using morpholino-mediated knockdown as well as overexpression of *adtrp1*, Wang et al. [46] showed that *adtrp1* regulates the expression of *tfpi* and consequently the specification of hemangioblasts and hematopoiesis. Potential mechanisms may involve TFPI interaction with glypican-3 and inhibition of the serine protease CD26 activity, which can lead to increased proliferation and migration of hematopoietic stem cells. Although considered to be independent biological processes, hematopoiesis and coagulation could be mechanistically connected through an ADTRP-TFPI regulatory axis.

#### 4.3.3. ADTRP-TFPI Interaction and Hemostasis/Thrombosis Defects 

Our genetic mouse model of Adtrp global deletion [39] revealed that Adtrp^−/−^ mice did not display major hemostasis defects, but we observed increased congestion and bleeding/extravascular fibrin, which associated with decreased TFPI antigen and anticoagulant activity, measured both in the plasma and in the lung. Other organs (liver, kidney) showed similar signs of congestion, bleeding and thrombosis, as well as signs of necrosis (kidney tubules) indicating tissue damage due to ischemia/reperfusion injury that may occur post-thrombosis (Lupu lab, unpublished data).

Although *Adtrp* deficiency in the mouse did not reduce *Tfpi* expression, it did affect TFPI-dependent lung-associated anticoagulant activity; therefore, we suggest that ADTRP may regulate the membrane location and anticoagulant potential of TFPI in EC in vivo similar to the described in vitro effects [28,39].

## 5. Role of ADTRP in Cardiovascular Health and Disease

### 5.1. rs6903956 in ADTRP Is a Novel Susceptibility Locus for CAD

As reviewed by Wang and colleagues [38], CAD, a leading cause of morbidity and mortality, is a complex disease caused by both genetic and environmental factors and their interactions. Risk factors for CAD include smoking, diabetes, hypertension, obesity, and alcohol. Among genetic factors, genome-wide association studies (GWAS) have identified single nucleotide polymorphisms (SNPs) on several chromosomes that are associated with the risk of CAD or its major complication, MI [47]. The association between SNPs on 9p21 and CAD/MI is the most robustly replicated finding in every ethnic population tested [48]. 

GWAS analysis in a Chinese Han cohort [38] revealed a new association between SNP rs6903956 in C6orf105 (ADTRP) on chromosome 6p24.1 and CAD, whereby the A allele of rs6903956 is significantly associated with the risk of MI, even after adjusting for gender, age, hypertension, diabetes, smoking, and atherogenic lipid levels. SNP rs6903956 associates with decreased expression of *ADTRP*, which could be partially responsible for pathogenesis in CAD/MI. SNP rs6903956 within the ADTRP gene is also significantly associated with CAD in ethnic groups in Singapore independently of conventional risk factors [49].

Guo and colleagues [50] analyzed arteriographies for coronary atherosclerosis, whose severity was defined by Gensini’s Score System and counts of diseased vessels. They describe the genotype distribution of the rs6903956 polymorphism and its association with the angiographical characteristics of coronary atherosclerosis risk. A stratification analysis among high Gensini score subjects revealed that male subjects and smoking subjects had a higher frequency of the rs6903956 heterozygous mutant.

A positive association between CAD risk variants was demonstrated for ADTRP and melanoma inhibitory activity protein 3 (MIA3)/transport and Golgi organization protein 1 (TANGO1), whereby ADTRP up-regulates the expression of *MIA3/TANGO1* through positive regulation of *PIK3R3* and activation of AKT signaling [51]. SNP rs17465637 in *MIA3/TANGO1* is a significant CAD risk variant [52]. MIA3/TANGO1 is involved in the export of collagen VII and ApoB from the endoplasmic reticulum [53] and the secretion of multiple types of collagens [54]. Whereas excessive collagen accumulation may lead to stenosis in CAD, low collagen levels could weaken the plaque causing rupture and leading to MI. Luo and colleagues [51] suggested that the low expression of *ADTRP* associated with rs6903956 may be linked to a higher risk of CAD/MI through MIA3/TANGO1- dependent increase of ApoB that transports pro-atherogenic cholesterol. These ideas are contradictory though, since reduction of ADTRP expression correlates with decreased MIA3/TANGO, whereas increased ApoB export requires higher, not diminished levels of MIA3/TANGO. 

All the data reporting associations between SNP rs6903956 in ADTRP and CAD are of potential interest, but significantly more experimental work especially in animal models will be necessary to prove causality for this mutation(s) and decipher the mechanisms that link low ADTRP expression to the increased risk of CAD/MI.

### 5.2. ADTRP Up-Regulation May Be Beneficial for Cardiovascular Function

ADTRP could be a pro-survival factor, as revealed by microarrays for *ADTRP* knockdown in human HepG2 and EC, where up-regulation of seven histone genes, down-regulation of cell cycle genes *CCND1*, *CDK4*, and *CDKN1A*, and up-regulation of apoptosis genes *CASP7* and *PDCD2* were found [55]. Our own qPCR data on newborn skin from Adtrp^−/−^ mice vs. WT (partial data published [39]) also showed significant up-regulation of several apoptosis-promoting genes: Caspases 3 and 4, Pdcd5 (programmed cell death 5), Ecscr (EC surface expressed chemotaxis and apoptosis regulator). Altogether, these data suggest that ADTRP may promote cell proliferation and inhibit apoptosis, which could be beneficial against the development of CAD/MI.

Another beneficial effect of increasing ADTRP level could relate to the effects of androgen. Low cellular ADTRP may increase the risk of CAD/MI by affecting functions of EC that are relevant to the development of atherosclerosis, such as increasing oxidized-LDL-mediated monocyte adhesion and transmigration through EC [42]. Androgen inhibits monocytes adhesion to EC and blocks their transmigration in a manner dependent on *ADTRP* expression [42]. Although studied for a long time, there is still no consensus regarding the relationship between androgen and CVD. Low androgen levels are associated with insulin resistance, diabetes and metabolic syndrome, atherosclerosis, cardiovascular risk, stroke, and cardiovascular mortality in men [56], but the causality of these associations remains to be proven. We speculate that if androgens exert protective roles against the risk of CAD/MI and thrombosis, they may do so at least in part through up-regulating the expression of ADTRP [28,42].

Besides androgen, Chinetti-Gbaguidi and colleagues showed that ADTRP is also transcriptionally controlled by PPAR- in monocytes/macrophages and up-regulated by PPAR- agonists in human atherosclerotic plaques [37]. Macrophages play crucial roles in the pathogenesis of atherosclerosis by controlling inflammation and cholesterol trafficking, and they are mainly associated with unstable, lipid-rich and thin capped plaques, where they contribute to the highly inflammatory and pro-thrombotic milieu with high levels of TF and non-matching levels of TFPI [4,5,6,57]. Similar to ADTRP, PPAR- activation up-regulates TFPI expression in monocytes/macrophages both in vitro and in vivo, a process that could control the inflammatory and pro-thrombotic effects of TF-dependent pathway activation [58]. In turn, TFPI can regulate macrophage differentiation, inhibit proliferation, and prevent foam cells formation [59]. High ADTRP expression in the macrophage-rich areas of human atherosclerotic plaques [60] together with increased TFPI may have beneficial effects in promoting plaque stability. Ongoing work in our lab seeks to identify the precise localization of ADTRP within the plaque, as well as possible connections with TFPI and their reciprocal regulation in relation to plaques heterogeneity.

Interestingly, Ooi and colleagues reported measurement of ADTRP in the plasma, and found that CAD patients had lower ADTRP levels than controls [41]. Although statistically significant, the differences were marginal, and the assays lacked validation. Nevertheless, the authors advanced the idea that the level of ADTRP could reflect a protective function, with every 100 pg/mL increase in ADTRP corresponding to a reduction of CAD risk by 9% [41], and suggested that plasma ADTRP may be a novel independent biomarker for CAD. However, the authors made no attempt to identify the cellular origin(s) of the presumably soluble ADTRP, nor address what a potential release of ADTRP could actually signify. If lower levels of circulating ADTRP correlate with low *ADTRP* expression in EC and/or monocytes, this may reflect cellular dysfunction, which would indeed support the development of CAD/MI, but this needs to be experimentally demonstrated. Knowing that plasma TFPI level is significantly higher in CVD and other pathologies, including COVID-19 [3], where TFPI increase is universally accepted to reflect endotheliopathy, it is crucially important to correlate the plasma levels of ADTRP with those of TFPI in the same sample groups. Because ADTRP is a transmembrane protein with location in lipid rafts/caveolae, it is hard to envision how it could be released from the cells. We can speculate that a circulating pool of ADTRP may reflect the release of ADTRP- containing microvesicles/exosomes from either EC or blood cells, but to demonstrate this, solid experimental data will be required. 

### 5.3. Role of ADTRP in Vascular Development and Homeostasis

Using mouse and zebrafish models of Adtrp genetic inhibition, we showed that ADTRP deficiency leads to leaky vessels, edema, and hemorrhage involving defective EC junctions, degradation of extracellular matrix (ECM) and local inflammation [39]. Abnormal vascular remodeling and angiogenesis play a significant part in the pathogenesis of atherosclerosis, hypertension and tumors, among others. 

Defective vascular features associated with ADTRP deficiency include abnormal dilation, tortuosity and branching, increased permeability/microhemorrhage, EC junction defects (decreased VE-cadherin and claudin-5), loose perivascular cell coverage and degraded ECM [39]. Neo-/perinatal mortality of ~35% in Adtrp^−/−^ mice correlated with the degree of phenotype severity. In independently generated Adtrp mutant mice, another group [40] found that mice lacking ADTRP developed normally with the expected Mendelian ratio. The discrepancy between our mice and theirs can be attributed to different strategies applied to generate ADTRP-deficient mice. Whereas we specifically deleted ~37 kb of the Adtrp gene between exons 2 and 6, Erikci Ertunc and colleagues [40] targeted only exon 2 using CRISPR/Cas9 technology, which resulted in the removal of 13-bases from the Adtrp gene locus. Further studies are warranted to understand the differences in these two mouse strains. Mechanistically, we demonstrated that the vascular defects were the consequence of significant temporal and spatial aberrant activation of the canonical Wnt pathway. Since we found that ADTRP acts downstream of LDL receptor-related protein-6 phosphorylation but upstream of glycogen synthase kinase-3β, we speculate that ADTRP may be binding to Axin-2 and/or interfere with the signalosome formation, similarly to the way that ADTRP regulates TFPI localization in EC lipid rafts/caveolae [28]. Accordingly, the palmitoylation-deficient ADTRP_mut_ described above failed to down-regulate the Wnt response (Lupu lab, unpublished data), indicating that the membrane localization of ADTRP is critical for its role in the inhibition of Wnt signaling.

Wnt-target genes consistently up-regulated in ADTRP-deficient mice and zebrafish include MMP-9 and IL-8, the latter being also a mediator of abnormal angiogenesis and increased MMP9 expression [61]. One prominent feature associated with ADTRP deficiency was the strikingly high accumulation of mast cells, whose degranulation products, including MMP-9 and other proteases could contribute to the inflammation and vascular defects observed. Since MMP-9 and IL-8 are known to contribute to the development of human atherosclerotic plaques [60], we can speculate that, through its prominent role in vascular homeostasis ADTRP may be critically involved in pathologies that feature disruption of the vascular wall, such as CAD/MI, DVT/VTE, sepsis, and cancer.

## 6. Function of ADTRP as an Endogenous Membrane Atypical Hydrolase 

Parsons and colleagues [35] have shown that ADTRP displays hydrolase activity. They used probes directed to identify Ser hydrolases and, to a lesser extent, Thr hydrolases, to perform activity-based protein profiling and identified AIG1 as a novel hydrolase enzyme. AIG1 has ~37% sequence homology with ADTRP. Therefore, the hydrolase activity was further characterized for both ADTRP and AIG1. Given that both are multi-pass transmembrane proteins, their enzymatic activity was assessed against various lipid substrates (e.g., lyso-phospholipids, neutral lipids, etc.). Both ADTRP and AIG1 efficiently hydrolyzed a novel class of lipids called fatty-acid esters of hydroxyl-fatty acids (FAHFAs). The hydrolase function depends on conserved Thr and His residues: T_47_ and H_131_ in ADTRP (Figure 1), T_43_ and H_134_ in AIG1. Hence, ADTRP and AIG1 are classified now as atypical Thr hydrolases of FAHFAs [35].

FAHFAs represent an endogenous class of lipids that are chemically composed of fatty acids and hydroxy fatty acids. In the past decade, palmitic acid esters of hydroxy stearic acids (PAHSAs) family of FAHFAs have gained attention because of their beneficial effect in diabetes and inflammatory pathologies. PAHSAs could improve glucose-insulin homeostasis in insulin-resistant mice fed a high-fat diet by stimulating the release of glucagon-like peptide 1 [62,63]. They also have a potent anti-inflammatory activity against lipopolysaccharide stimulation, characterized by reduced cytokine production and dendritic cell activation [62,64]. Oral administration of 5-PAHSA and 9-PAHSA protected mice against dextran sulfate sodium-induced colitis, a well-characterized model of ulcerative colitis, by modulating the innate and adaptive immune responses in the gut [65]. In a recent comprehensive review, Brejchova and colleagues analyzed the current data on FAHFAs and addressed their role as key lipid messengers that are able to manage immuno-metabolic and inflammatory processes [66]. As ADTRP was shown to specifically hydrolyze FAHFAs, including 5-PAHSA and 9-PAHSA in cell culture, it is important to understand the physiological role of ADTRP in regulating endogenous FAHFAs in vivo. 

In a recent study, Erikci Ertunc and colleagues used a combination of genetic loss-of-function and chemical approaches to demonstrate that ADTRP and AIG1 are major contributors to endogenous FAHFA hydrolysis [40]. They used global *Aig1*, *Adtrp*, and double knockout (DKO) mice and a small molecule inhibitor (ABD-110207) of ADTRP and AIG1 to show that both proteins are responsible for regulating FAHFA hydrolysis primarily in the adipose tissue. They also found that ADTRP was the major driver of 9-PAHSA hydrolysis in the liver, whereas ADTRP and AIG1 had an additive effect in brown adipose tissue and kidney. Interestingly, tissues from DKO mice showed decreased hydrolytic activity towards 9-PAHSA added exogenously but no change in the overall endogenous FAHFA levels. In parallel, a significant residual 9-PAHSA hydrolytic activity was observed in the liver and kidney of DKO mice [40]. Together, these data indicate that other endogenous enzymes, such as carboxyl ester lipase [67] can compensate for loss of ADTRP and/or AIG1 to hydrolyze FAHFAs in vivo. 

Intriguingly, the loss of ADTRP and/or AIG1 failed to protect the mice against high-fat diet-induced obesity and metabolic dysregulation, despite elevated FAHFA levels in adipose tissue [40]. The lack of protective effect could be attributed to: (i) ADTRP and/or AIG1 deficient mice do not achieve high enough levels of FAHFA as when FAHFAs are administered exogenously [40,62,63]; (ii) differential expression and tissue-specific hydrolytic activity of ADTRP and AIG1; (iii) variable bioavailability of exogenously administered FAHFAs in different organs, like the liver which has high metabolic relevance; and (iv) the compensatory role of other hydrolases. 

Collectively, these studies highlight the importance of ADTRP as an endogenous FAHFA hydrolase and its potential as a drug target. Considering that ADTRP and AIG1 are androgen inducible genes, it will be interesting to investigate sex-based differences in FAHFA regulation and consecutive effects on high-fat diet-induced metabolic disorders. It would be of great interest to determine whether the hydrolase activity of ADTRP may play a role in the regulation of TFPI anticoagulant function in EC. Further studies are also required to elucidate the role of tissue-specific ADTRP mediated FAHFA hydrolysis in the context of inflammatory responses and CVD.

## 7. Role of ADTRP in Stem Cells Differentiation 

Deficiency of ADTRP in oral cleft syndrome [68] and craniosynostosis [69] suggests that ADTRP may play significant roles in the epithelial to mesenchymal transition during palatogenesis, and MSC differentiation into chondrocytes and osteocytes. ADTRP (C6ORF105) was one of the highly significant genes selectively expressed in human bone marrow MSC (BM-MSC) as compared to fibroblasts, osteoblasts, chondrocytes and adipocytes, and most of these MSC-characteristic genes were down-regulated 24 h after induction of differentiation [36]. 

**Figure 6:**
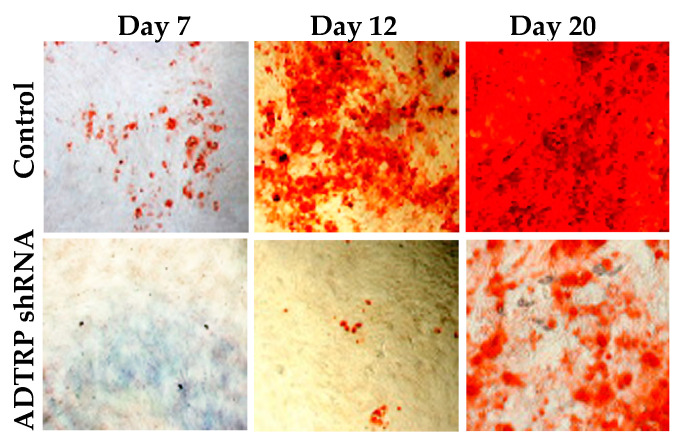
Role of ADTRP in bone marrow mesenchymal stem cells (BM-MSC) differentiation. Human BM-MSC (Texas A&M College of Medicine’s Institute for Regenerative Medicine, College Station, TX, USA) were induced with osteogenic medium (alpha-MEM with 16.5% FBS, 10 nM dexamethasone, 50 µM L-ascorbic acid 2-phosphate sesquimagnesium salt and 20 mM β-glycerolphosphate) and assessed for osteogenic differentiation by Alizarin S staining. Endogenous ADTRP was silenced before and every 3 days during the osteogenic induction and differentiation, by using RNA interference (ADTRP shRNA) as described [28].

Our own experiments addressing the role of ADTRP in human BM-MSC also showed decreased ADTRP mRNA expression after induction with osteogenesis medium. Comparison between Control BM-MSC and cells where *ADTRP* was down-regulated using shRNA shows significant impairment of mineralization in ADTRP-deficient cells, illustrated by Alizarin Red staining (Figure 6).

In contrast to the human BM-MSC data, we did not observe any obvious bone/cartilage/oral cleft defects in our Adtrp^−/−^ mice. However, experiments like bone densitometry, alcian blue staining in embryos and neonates, and challenge models like bone-fracture and healing models are yet to be employed to properly characterize the role of ADTRP in MSC differentiation/bone formation in mice.

It is known that androgens stimulate MSC differentiation into osteo-muscular rather than fat tissue through mechanisms involving AR and inhibition of the adipogenic factor PPAR-γ. AR controls pluripotency transcription factors and acts as negative regulator of signaling pathways that maintain the stem cell state, such as the PI3K/AKT [70]. The decrease of ADTRP expression in MSC after induction of osteogenic differentiation described above does not correlate well with the effect of androgen/AR but would strengthen the Wnt/*β*-catenin activation, which is critically important for BM-MSC differentiation in response to androgen [71]. 

## 8. Conclusions and Future Perspectives

**Figure 7:**
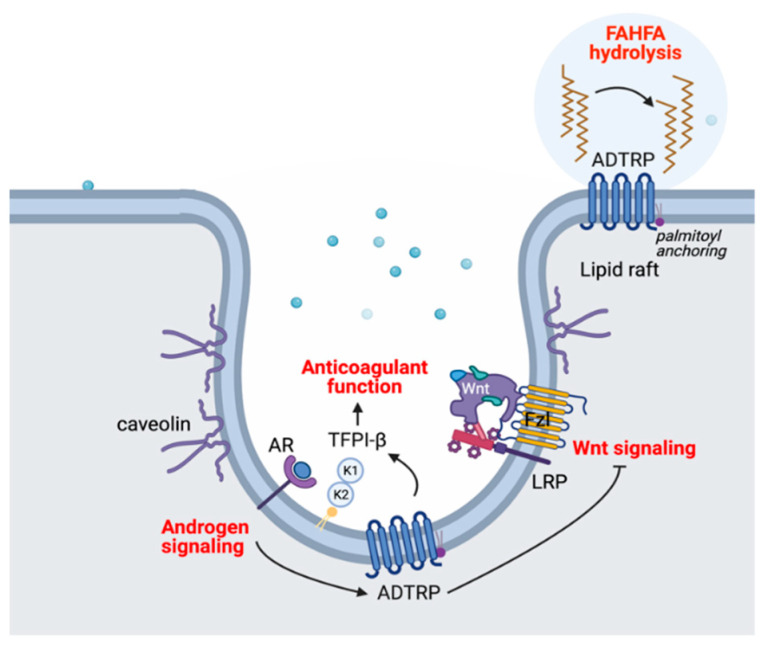
Proposed model for some of the cellular actions of ADTRP. In EC, ADTRP co-localizes with both the GPI-anchored TFPIβ and caveolin-1. Palmitoylation of ADTRP supports its membrane localization in lipid rafts/caveolae. Androgen signaling through androgen receptor (AR) up-regulates the expression of ADTRP, which in turn enhances TFPI clustering and activity thus increasing its anticoagulant function, and inhibits Wnt signaling. ADTRP regulates FAHFAs hydrolysis in the adipose tissue, liver and kidney. K1 and K2: Kunitz-1 and 2 domains of TFPIβ; Fzl: frizzled receptor; LRP: LDL receptor-related protein.

While a significant amount of information has accumulated within the decade since its discovery, the primary function of ADTRP remains elusive. So far, data are hinting towards multiple roles, some of which are depicted in Figure 7. 

ADTRP is necessary for maintaining cell and tissue homeostasis, including regulation of TFPI-dependent anticoagulant activity, preserving vascular integrity and normal cardiovascular function, prevention of unwanted Wnt signaling, and stem cell differentiation. On the other hand, reduced ADTRP hydrolase activity may help increase the level of the metabolically beneficial FAHFAs. Apparently discrepant roles could arise from differences in organ-specific expression and possibly distinct mechanisms of regulation of ADTRP. Despite advancements, significant mechanistic work is still necessary to discriminate between the primary and secondary functional roles of ADTRP. 

Since ADTRP has a wide tissue expression and it is evolutionary conserved having orthologs in all classes of vertebrates, it is expected to play essential functions. However, ADTRP gene deletion in mice was compatible with survival, suggesting the existence of compensatory mechanisms. Structure-function studies may help identify the primary role of ADTRP. Proteins with six transmembrane spanning domains have a variety of functions, including enzymatic activities, membrane transporters, and channels. Membrane anchoring of ADTRP suggests a potential role as membrane microdomain organizer, which may integrate some of the described cell surface activities, such as the modulation of TFPI-dependent anticoagulant function, Wnt and androgen signaling, and hydrolysis of membrane lipids (Figure 7). 

Future cell/tissue-specific gain and loss of function studies in models of metabolic disorders, coagulation/thrombosis, and atherosclerosis, coupled with in depth multi-omics analysis will be required to unveil the primary function of ADTRP and to understand its main involvement in the mechanism of diseases. 

## Data Availability

The original data presented in this review are available on request from the corresponding author.
